# Evaluation of the deeplex Myc-TB-targeted next-generation sequencing assay for mycobacterial identification and drug resistance detection

**DOI:** 10.1128/jcm.00082-26

**Published:** 2026-05-29

**Authors:** Kin Israel Notarte, Kristine Allen, Derek T. Armstrong, Nicole Parrish

**Affiliations:** 1 Department of Pathology, The Johns Hopkins Hospital 588543, Baltimore, Maryland, USA; The University of North Carolina at Chapel Hill School of Medicine, Chapel Hill, North Carolina, USA

**Keywords:** *Mycobacterium tuberculosis*, nontuberculous mycobacteria, targeted next-generation sequencing, genotypic testing, drug resistance, molecular diagnostics

## Abstract

Accurate differentiation of *Mycobacterium tuberculosis* complex (MTBC) and nontuberculous mycobacteria (NTM), together with early detection of drug resistance, is essential for guiding therapy and infection control. Targeted next-generation sequencing assays, such as the Deeplex Myc-TB (GenoScreen, Lille, France), allow simultaneous identification of species and detection of drug-resistance mutations. We evaluated Deeplex Myc-TB on 304 primary, culture-positive mycobacterial growth indicator tube (MGIT) broths collected between May 2024 and March 2025. The assay generated determinate results for 273 isolates, comprising 29 MTBC and 244 NTM, with 100% agreement for MTBC identification, and 98.8% for NTM. MTBC identification was performed using GeneXpert MTB/RIF, whereas phenotypic drug susceptibility testing (pDST) was performed using either the Sensititre MYCOTB broth microdilution method, or the MGIT-based panel. Among the 29 unique MTBC isolates, 18 (62.1%) had no detectable drug-resistance mutations (DRMs). Phenotypic drug susceptibility testing confirmed susceptibility to all tested drugs for these 18 isolates, resulting in 100% concordance with Deeplex-predicted susceptibility, and no evidence of heteroresistance. The remaining 11 isolates (37.9%) carried at least one DRM, including seven with canonical resistance-conferring mutations. For isolates with canonical DRMs and available CLSI breakpoints, genotypic calls and pDST were concordant, and one isolate met the criteria for multidrug-resistant tuberculosis. All 244 NTM isolates had prior matrix-assisted laser desorption/ionization time-of-flight mass spectrometry (MALDI-TOF MS) identification; Deeplex confirmed these identifications and provided subspecies-level resolution within the *Mycobacterium avium* complex and *Mycobacterium abscessus*. These findings demonstrate the strong diagnostic potential of Deeplex Myc-TB for high-resolution MTBC resistance profiling and precise NTM species and subspecies identification. Integrating targeted sequencing into routine workflows can improve clinical decision-making, support early treatment optimization, and strengthen molecular surveillance of mycobacterial diseases.

**IMPORTANCE:**

Rapid and accurate identification of mycobacteria and detection of drug resistance are essential for timely patient management and public health control of tuberculosis and nontuberculous mycobacterial infections. However, routine diagnostics often rely on multiple sequential assays, which can delay comprehensive results. In this study, we demonstrate that targeted next-generation sequencing using Deeplex Myc-TB can provide high-resolution species identification and clinically meaningful drug-resistance information in a single workflow within a U.S. clinical laboratory setting. The assay showed complete concordance with GeneXpert MTB/RIF for identification of the *Mycobacterium tuberculosis* complex and produced genotypic resistance predictions consistent with phenotypic drug susceptibility testing, where interpretive criteria were available. For nontuberculous mycobacteria, the assay confirmed MALDI-TOF MS identifications and enabled subspecies-level discrimination within clinically important groups, including the *Mycobacterium avium* complex and *Mycobacterium abscessus*. These findings support the integration of targeted sequencing into routine mycobacterial diagnostics to streamline laboratory workflows, improve the timeliness and clinical relevance of reporting, and enhance molecular surveillance efforts.

## INTRODUCTION

The *Mycobacterium tuberculosis* complex (MTBC) remains a leading cause of morbidity and mortality worldwide; however, diagnostic workflows are limited by the pathogen’s slow and fastidious growth (1). Culture-based phenotypic drug susceptibility testing (pDST), the long-standing reference standard, often requires about 30 days for results (2), delaying treatment initiation and sustaining transmission. Molecular methods have partly addressed these limitations, with assays such as the Xpert MTB/RIF assay (Cepheid, Sunnyvale, CA), enabling rapid detection of MTBC and rifampin resistance within hours (3). However, its scope is restricted to a single resistance target, and it cannot provide comprehensive resistance profiling or identify nontuberculous mycobacteria (NTM) (4, 5).

NTM represent a heterogeneous group of over 200 species with diverse clinical implications (6). While some act as environmental colonizers, others cause severe pulmonary or disseminated disease (7). Treatment regimens vary considerably between species and subspecies, underscoring the importance of accurate and timely identification (7, 8). However, conventional approaches often lack the speed and discriminatory power needed for clinical decision-making, leading to diagnostic uncertainty and inappropriate therapy (9).

The emergence of drug-resistant TB further highlights the need for advanced diagnostics. The World Health Organization (WHO) Global Tuberculosis Report estimated approximately 410,000 incident cases of multidrug-resistant or rifampin-resistant TB (MDR/RR-TB) globally in 2022 (1), including a substantial subset of pre-extensively drug-resistant TB (pre-XDR-TB) or extensively drug-resistant TB (XDR-TB) (10). Although the U.S. has a lower overall TB burden compared to high-incidence regions, it faces unique diagnostic challenges (11). Drug-resistant TB remains a public health threat in immigrant and immunocompromised populations (12, 13); although clinically significant, NTM infections are increasingly recognized across the country, particularly in individuals with chronic lung disease (14, 15). Rapid and accurate tools for both MTBC resistance detection and NTM species identification are therefore essential to improve patient outcomes and inform infection control strategies in the U.S. healthcare setting.

Targeted next-generation sequencing (tNGS) offers a practical solution by identifying resistance-associated loci across the mycobacterial genome. This approach provides high concordance with pDST, enables species-level identification of NTM, and generates actionable insights to support individualized therapy (2, 16, 17). Among available platforms, the Deeplex Myc-TB assay is one of the most advanced applications of tNGS. It has now been evaluated in national surveys and reference laboratories across Africa, Asia, and Europe, using both direct sputum and cultured isolates, and has shown high concordance with pDST for first- and second-line drugs, reliable detection of minority resistant variants, and robust performance in high–drug-resistant-burden settings (2, 18–21). Additionally, the platform simultaneously predicts resistance to 13 anti-tuberculosis drugs or drug classes, performs MTBC genotyping, and identifies NTM species, all through a secure, cloud-based pipeline (2, 16, 17). Each run incorporates built-in quality control measures, including positive and negative controls to monitor amplification performance and detect contamination, as well as internal controls to verify sequencing depth and coverage uniformity, ensuring the reliability of each assay result (22). Recent WHO evaluations have recognized Deeplex Myc-TB as the only tNGS assay meeting performance criteria for all drugs evaluated, while other tNGS assays have been recommended for specific subsets of anti-tuberculosis drugs, including AmPORE-TB for rifampin (RIF), isoniazid (INH), fluoroquinolones (FQs), linezolid (LZD), amikacin (AMK), and streptomycin (STR), and TBseq for ethambutol (EMB) (23).

Although Deeplex Myc-TB was developed for use on both direct respiratory specimens and cultured isolates, this study focuses on its implementation using culture-positive MGIT broth specimens within a routine clinical laboratory setting. Here, we describe the integration of Deeplex Myc-TB into our clinical mycobacteriology laboratory and evaluate its diagnostic performance under real-world conditions. Specifically, we assess concordance between Deeplex-based MTBC identification and drug-resistance predictions and those obtained with GeneXpert MTB/RIF and pDST and examine agreement between Deeplex and MALDI-TOF MS for NTM identification. This evaluation provides pragmatic data on the performance and integration of a targeted next-generation sequencing assay into mycobacterial diagnostics at a U.S. tertiary-care center.

## MATERIALS AND METHODS

### Clinical specimens and laboratory workflow

We evaluated acid-fast bacilli (AFB)–positive cultures from an automated culture detection system (MGIT-960, Becton Dickinson, Sparks, MD) at the Johns Hopkins Clinical Mycobacteriology Laboratory from May 2024 through March 2025. MGITs that flagged positive by the instrument underwent AFB microscopy to confirm the presence of acid-fast bacilli prior to downstream testing. As part of routine clinical care, AFB-positive MGITs were tested by GeneXpert MTB/RIF (Cepheid) (24) to detect *Mycobacterium tuberculosis* complex (MTBC). All MGIT cultures that tested positive for MTBC by GeneXpert MTB/RIF were included for Deeplex Myc-TB testing to assess concordance in MTBC identification, rifampin resistance prediction, and phenotypic drug susceptibility testing (pDST). Phenotypic DST for MTBC was performed using either the commercially available lyophilized Sensititre MYCOTB broth microdilution panel or a custom frozen panel containing the novel drugs bedaquiline (BDQ), linezolid (LZD), pretomanid (Pa), and clofazimine (CFZ) (Thermo Fisher Scientific, Lenexa, Kansas) (25, 26). Panel selection was based on plate availability at the time of testing; a custom panel was used when testing required inclusion of newer or novel anti-tuberculosis agents not present on the commercially available panel. MGIT-based testing (27) for isoniazid (INH), rifampin (RIF), ethambutol (EMB), and pyrazinamide (PZA) was performed where indicated and interpreted according to the Clinical and Laboratory Standards Institute (CLSI) guidelines, where breakpoints were available (28).

AFB-positive MGITs that were confirmed negative for MTBC by GeneXpert were worked up as nontuberculous mycobacteria (NTM). These NTM isolates underwent identification by matrix-assisted laser desorption/ionization time-of-flight mass spectrometry (MALDI-TOF MS) (Bruker, Billerica, MA) (29). Isolates for identification by MALDI-TOF MS were extracted using adaptive focused acoustics and analyzed using the Bruker BioTyper software and mycobacterial reference database (29). MALDI-TOF MS identifications with a log score >1.8 were subsequently included for Deeplex Myc-TB testing to compare NTM species calls between MALDI-TOF MS and tNGS.

### Deeplex Myc-TB assay

For each culture selected for sequencing, a 1.0-mL aliquot of broth was processed using the Deeplex Myc-TB kit (23). DNA extraction was performed on a KingFisher Duo instrument (ThermoFisher Scientific), followed by multiplex PCR amplification with kit-supplied primers, library preparation, and sequencing on an Illumina iSeq platform. Sequencing data were analyzed with the manufacturer’s secure, web-based application, which aligns reads to a curated mycobacterial reference database and reports MTBC lineage, NTM species/subspecies, and drug-resistance mutations for MTBC (22, 30). The results were considered acceptable if they achieved a mean coverage depth >20% across targeted loci, 100% identity for species- and subspecies-level calls, and successful performance of all five internal quality controls included in each run (positive and negative PCR controls, positive and negative extraction controls, and an internal control). The >20% mean coverage depth threshold was selected based on internal laboratory quality-control criteria to ensure reliable target detection and variant calling; samples not meeting this threshold were classified as indeterminate and excluded from concordance analyses.

## RESULTS

A total of 304 cultures were tested using the Deeplex Myc-TB assay between May 2024 and March 2025. Of these, 31 (10.2%) were excluded because the results were not interpretable, most commonly due to insufficient sequencing coverage or unexpected mixed mycobacterial detections by Deeplex Myc-TB that were not corroborated by culture, which were considered contamination. The remaining 273 (89.8%) isolates, comprising 29 MTBC and 244 NTM, met the quality criteria specified in the Methods section and were included in the final analysis.

All 29 culture-positive isolates that were GeneXpert-positive for MTBC were also identified as MTBC by Deeplex Myc-TB, demonstrating 100% agreement between the two assays for MTBC identification. Among the 29 MTBC isolates, 18 (62.1%) had no Deeplex-detected drug-resistance mutations (DRMs) across all targeted genes. Phenotypic DST for these 18 isolates confirmed susceptibility to all tested drugs, with 100% concordance with Deeplex-predicted susceptibility. No heteroresistance was detected among the MTBC isolates, as resistance-associated loci showed uniform wild-type sequences.

Table 1 summarizes the 11 MTBC isolates (37.9%) with Deeplex-detected genotypic mutations, categorized as known or uncertain significance according to the WHO Catalog of mutations in MTBC (31). Seven isolates (24.1%) carried at least one mutation of known significance, seven (24.1%) carried at least one mutation of uncertain significance, and three (10.3%) carried mutations in both categories. Where phenotypic testing and interpretive criteria were available, isolates harboring only variants of unknown significance were phenotypically susceptible, supporting that these variants were not associated with phenotypic resistance in this data set.

**TABLE 1:** Concordance between Deeplex-predicted resistance and phenotypic DST with CLSI category assignment^*b*^

Isolate no.	Drug	^*a*^Genotypic mutation		^*c*^Phenotypic DST	Phenotypic call
		Known significance	Uncertain significance		
1	INH	*inhA* (S94A)		MGIT (INH): 0.1 µg/mL (R), 0.4 µg/mL (S)	Low-level resistance (INH)
3	RIF		*rpoB* (E291D)	MIC (RIF): 0.25 µg/mL (S)	Susceptible (RIF)
	FQ		*gyrA* (V55M)	MIC (OFL): 0.5 µg/mL MIC (MXF): 0.12 µg/mL	No CLSI breakpoint (OFL, MXF); not interpreted
5	ETH		*ethA* (S266R)	MIC (ETH): 0.6 µg/mL	^*d*^No CLSI breakpoint (ETH); not interpreted
6	INH		*ahpC* (g-123a)	MIC (INH): 0.03 µg/mL (S)	Susceptible (INH)
	RIF		*rpoB* (G217S)	MIC (RIF): 0.25 µg/mL (S)	Susceptible (RIF)
7	PZA	*pncA* (H57D)		MGIT (PZA): 100 µg/mL (R)	Confirmed resistance (PZA)
9	INH	*fabG1* (c-15t)		MIC (INH): 0.5 µg/mL (R)	High-level resistance (INH)
	ETH	*fabG1* (c-15t)		MIC (ETH): 4 µg/mL	^*d*^No CLSI breakpoint (ETH); not interpreted
13	INH	*fabG1* (c-15t)		MIC (INH): 0.5 µg/mL (R)	High-level resistance (INH)
			*katG* (G186N)		
	ETH	*fabG1* (c-15t)		MIC (ETH): 2 µg/mL	^*d*^No CLSI breakpoint (ETH); not interpreted
15	RIF	*rpoB* (S450L)		MIC (RIF): >8 µg/ml (R)	Confirmed resistance (RIF)
			*rpoB* (V496A)		
	INH	*katG* (S315T)		MIC (INH): 3.2 µg/mL (R)	High-level resistance (INH)
	EMB	*embB* (M306I)		MIC (EMB): 10 µg/mL (R)	Confirmed resistance (EMB)
	STM	*rpsL* (K43R)		Not tested	Frozen panel excludes STM; not interpreted
	ETH		*ethA* (delA)	Not tested	Frozen panel excludes ETH; not interpreted
17	INH		*ahpC* (g-142a)	MIC (INH): 0.03 µg/mL (S)	Susceptible (INH)
	STM	*gidB* (delG)		MIC (STM): 4 µg/mL	No CLSI breakpoint (STM)
18	LZD		*rrl* (G2399A)	Not tested	Lyophilized panel excludes LZD
27	STM	*gidB* (del)		Not tested	Frozen panel excludes STM

^*a*^ Genotypic mutation significance was assigned according to the WHO catalog of mutations in MTBC [31].

^*b*^ RIF, Rifampin; INH, Isoniazid; PZA, Pyrazinamide; EMB, Ethambutol; FQ, Fluoroquinolone; OFL, Ofloxacin; MXF, Moxifloxacin; LZD, Linezolid; STM, Streptomycin; ETH, Ethionamide; DRMs, Drug-resistance mutations; DST, Drug susceptibility testing.

^*c*^ MICs were determined using either the Sensititre MYCOTB panel or frozen custom MIC panel; MGIT was used for INH and PZA where indicated.

^*d*^ Although no CLSI MIC breakpoint is defined for ETH on this panel, mutations in* inhA* or *fabG1* are typically associated with ETH resistance; hence, isolates carrying these mutations could likely represent ETH-resistant strains.

For isolates with known-significance DRMs where phenotypic breakpoints were available, genotypic and phenotypic calls were concordant. INH resistance was confirmed with mutations in the following genes: *inhA* (S94A), *katG* (S315T), and/or *fabG1* (c-15t). RIF resistance was confirmed with mutations in *rpoB* (S450L); EMB resistance with mutations in *embB* (M306I); and PZA resistance with mutations in *pncA* (H57D) (Table 1). Four isolates were mono-resistant based on Deeplex predictions (three INH-resistant and one PZA-resistant), all concordant with pDST. One isolate (isolate 15) met the criteria for multidrug-resistant tuberculosis (MDR-TB), with resistance to RIF and INH confirmed by both Deeplex and pDST; this isolate was also EMB-resistant by both methods. Isolate 15 additionally carried mutations in *rpsL* (K43R) and *ethA* (delA). Phenotypic DST for streptomycin and ethionamide (ETH) was not available for this isolate. No isolates met the criteria for pre-XDR or XDR.

Several additional Deeplex-flagged mutations were classified as of uncertain significance and/or lacked phenotypic interpretive criteria on the platform used. These included variants in *rpoB* (E291D, G217S, and V496A), *gyrA* (V55M), *ahpC* (g-123a and g-142a), *katG* (G186N), *rrl* (G2399A), *ethA* (S266R), and *gidB* (delG/del). As shown in Table 1, for variants of unknown significance with available breakpoints (e.g., *rpoB* E291D and *rpoB* G217S), MICs remained within the susceptible range (RIF MIC: 0.25 µg/mL), and categorical interpretations remained susceptible. For variants where CLSI breakpoints were not defined on the test platform (e.g., FQ and ETH) or where testing was not performed (LZD), the results could not be categorized despite the reported MICs or were not available and therefore did not change categorical assignment. Two isolates carried variants of unknown significance alongside canonical DRMs: isolate 13 had a *katG* (G186N) in the setting of *fabG1* (c-15t), and isolate 15 had *rpoB* (V496A) in the setting of *rpoB* (S450L). In both cases, phenotypic resistance was consistent with the canonical, known-significance mutations, while the additional variants were classified as uncertain significance.

A total of 244 isolates identified as NTM by MALDI-TOF MS (valid score >1.8) were included in Deeplex Myc-TB testing. Among isolates with determinate Deeplex species calls, Deeplex Myc-TB and MALDI-TOF MS were concordant in 241/244 cases (98.8%) at the taxonomic level resolved by MALDI-TOF MS. The NTM distribution shown in Table 2 reflects Deeplex Myc-TB identifications among the 241 isolates with determinate Deeplex species calls. MAC predominated, comprising 164 of 241 isolates (68.0%). Within MAC, the most frequent taxa were *Mycobacterium intracellulare* subsp. *intracellulare* (62/241, 25.7%), *M. intracellulare* subsp. *chimaera* (48/241, 19.9%), and *M. avium* (40/241, 16.6%), followed by *Mycobacterium marseillense* (13/241, 5.4%) and *Mycobacterium colombiense* (1/241, 0.4%). The *M. abscessus* group accounted for 64 isolates (26.6%), including *M. abscessus* subsp. *abscessus* (54/241, 22.4%) and *M. abscessus* subsp. *massiliense* (10/241, 4.1%). The *M. fortuitum* complex represented five isolates (2.1%). Other less frequent species totaled eight isolates (3.3%) and included *Mycobacterium haemophilum* (2/241, 0.8%), *Mycobacterium lentiflavum* (2/241, 0.8%), *Mycobacterium xenopi* (2/241, 0.8%), *Mycobacterium marinum* (1/241, 0.4%), and *Mycobacterium neoaurum* (1/241, 0.4%).

**TABLE 2:** Distribution of NTM species correctly identified by Deeplex Myc-TB

Identified NTM	No. of isolates	% of Total (*N* = 241)
*Mycobacterium avium* complex (MAC)		
*M. avium*	40	16.6
*M. colombiense*	1	0.4
*M. marseillense*	13	5.4
*M.intracellulare* subsp*. chimaera*	48	19.9
*M. intracellulare* subsp. *intracellulare*	62	25.7
Subtotal (MAC)	164	68.0
*Mycobacterium fortuitum* complex		
*M. fortuitum* subsp. *acetamidolyticum/fortuitum*	5	2.1
Subtotal (Fortuitum complex)	5	2.1
*Mycobacterium abscessus* (MAB)		
*M. abscessus* subsp. *abscessus*	54	22.4
*M. abscessus* subsp. *massiliense*	10	4.1
Subtotal (MAB)	64	26.6
Other NTM species		
*M. haemophilum*	2	0.8
*M. lentiflavum*	2	0.8
*M. marinum*	1	0.4
*M. neoaurum*	1	0.4
*M. xenopi*	2	0.8
Subtotal (other species)	8	3.3
Total	241	100

Three NTM cultures showed discordant organism identification between the initial Deeplex Myc-TB run and MALDI-TOF MS. One culture was identified as *M. heckeshornense* by MALDI-TOF MS and as *M. abscessus* subsp. *abscessus* by Deeplex Myc-TB. Upon repeat Deeplex Myc-TB testing, no organism identification was obtained; the isolate was subsequently evaluated by 16S sequencing and identified as *M. heckeshornense* at 100% identity. The second culture was identified as *M. porcinum* by MALDI-TOF MS and as *M. intracellulare* subsp. *intracellulare* by Deeplex Myc-TB. Because the isolate was morphologically consistent with *M. porcinum* and exhibited a growth rate characteristic of this species, the MALDI-TOF MS identification was accepted, and repeat Deeplex Myc-TB testing was not performed. Similarly, a third NTM was identified as *Mycobacterium peregrinum* on MALDI-TOF MS but showed no identification on Deeplex Myc-TB. Colony morphology and growth were typical of this species; therefore, the MALDI-TOF MS was accepted, and Deeplex Myc-TB testing was not repeated.

## DISCUSSION

This study shows that Deeplex Myc-TB can support routine mycobacteriology testing by providing accurate MTBC genotyping together with high-resolution identification of nontuberculous mycobacteria.

Importantly, a subset of cultures produced indeterminate results and were excluded from the primary concordance analyses. Specifically, 31 of 304 cultures (10.2%) were excluded due to contamination (8), repeated tests due to run failures (13), insufficient sequencing coverage depth (8), or uninterpretable results (2). At our institution, Deeplex is implemented as a batched assay with an approximate 1-week turnaround time from culture positivity to result, which is shorter than the turnaround time for liquid-based pDST using the Sensititre MYCOTB panel and MGIT. However, because Deeplex validated at our institution is culture-dependent and run in batches, the results are still not available at the time of initial treatment decisions.

From a practical laboratory perspective, cultures yielding indeterminate Deeplex Myc-TB results are not used for clinical interpretation. When sufficient material is available, Deeplex testing would be repeated once. If repeat testing remains indeterminate or is not feasible, organism identification and drug susceptibility testing would proceed using standard phenotypic and conventional molecular methods in accordance with routine clinical laboratory practice. This workflow reflects the role of Deeplex as a rapid front-end molecular screen rather than a standalone diagnostic replacement.

All isolates evaluated in this study were derived from primary, culture-positive MGIT broths generated through routine clinical workflows rather than from single-colony subcultures. As such, the potential for mixed mycobacterial populations exists. In our laboratory, however, mixed cultures are uncommon, and no confirmed mixed MTBC or NTM populations were identified among isolates yielding determinate Deeplex Myc-TB results. Cultures with evidence of mixed or non-interpretable sequencing signals were classified as indeterminate and excluded from concordance analyses. These findings therefore reflect the low frequency of mixed cultures encountered in routine practice rather than a limitation of Deeplex Myc-TB performance.

In this cohort, culture-positive MTBC isolates without detectable resistance mutations were susceptible to all drugs on our phenotypic panel, in complete agreement with Deeplex predictions. When canonical resistance mutations were present, phenotypic resistance to INH, RIF, EMB, and PZA matched the genotypic calls. Taken together, these findings position Deeplex as a rapid front-end screen of MTBC cultures that identifies validated resistance mutations and flags likely susceptible cases for streamlined reporting.

Beyond canonical mutations, several isolates harbored uncharacterized variants in resistance-associated genes. In two isolates (13 and 15), *katG* (G186N) and *rpoB* (V496A), respectively, occurred together with canonical *fabG1* (c-15t) and *rpoB* (S450L) mutations. These showed high-level INH or RIF resistance, suggesting that such additional changes may modulate resistance phenotypes in some genetic backgrounds. By contrast, when uncharacterized variants were present without known drug-resistance mutations (DRMs), such as *rpoB* (E291D or G217S), *ahpC* (g-123a or g-142a), or *gyrA* (V55M), MICs remained within the susceptible range or could not be categorized because of missing breakpoints, and CLSI susceptibility categories were unchanged. Although these variants are not validated predictors of resistance, their detection by Deeplex supports epidemiologic surveillance and enables future reinterpretation as curated mutation catalogs and functional data expand; on their own, they should not prompt changes in therapy.

Operational factors also shaped resistance category assignment. For STM and ETH, CLSI MIC breakpoints were either not available on the phenotypic panel or the drugs were not tested, leading to indeterminate results that did not alter overall resistance categories. This occurred for ETH in isolates 5, 9, and 13, where ETH MICs were measured (0.6, 4, and 2 μg/mL, respectively) but could not be categorized by CLSI criteria on this panel. In addition, ETH was not tested for isolate 15; hence, a phenotypic ETH call could not be made despite the detection of an ETH-associated variant (*ethA* delA). Similarly, STM was not tested for isolates 15 and 27, precluding CLSI category assignment despite the detection of STM-associated variants (*rpsL* K43R in isolate 15 and *gidB* del in isolate 27). These constraints reflect limits of the phenotypic testing framework rather than sequencing.

In parallel, the Deeplex panel interrogates resistance-associated loci for several additional agents, including bedaquiline (BDQ), clofazimine (CFZ), linezolid (LZD), fluoroquinolones (FQs, including moxifloxacin), and aminoglycosides (e.g., amikacin, kanamycin, and streptomycin). These targets encompass key components of contemporary regimens such as BPaLM (bedaquiline, pretomanid, linezolid, and moxifloxacin), although pretomanid-specific markers are not included in the current assay. Because formal interpretive criteria are not defined for the custom Sensititre MYCOTB pDST panel used for these agents, the observed MIC ranges are provided in Table S1 to contextualize the phenotypic findings relative to the current WHO MGIT critical concentrations.

Across this isolate set, Sensititre testing showed relatively narrow MIC ranges for moxifloxacin (0.12–1.0 µg/mL), amikacin (0.12–0.5 µg/mL), kanamycin (0.6–2.5 µg/mL), and streptomycin (0.25–4.0 µg/mL) (Table S1). Deeplex analysis did not identify any WHO-validated resistance-conferring mutations for these agents; instead, only uncharacterized variants were observed, including *gyrA* (V55M) within the fluoroquinolone resistance-determining region, *gidB* insertions/deletions and *rpsL* (K43R) associated with streptomycin, and *rrl* (G2399A) for linezolid, none of which currently have WHO-based resistance interpretation (Table S1).

The absence of validated resistance-conferring mutations alongside MIC distributions that largely fall at or below the WHO critical concentrations is consistent with likely susceptibility to these agents in this isolate set, while also illustrating how Deeplex Myc-TB can identify uncharacterized variants that may become clinically relevant as resistance catalogs evolve. Further alignment between molecular targets, phenotypic MIC panels, and interpretive criteria would strengthen the integration of molecular and phenotypic data in clinical decision-making.

For NTM, MALDI-TOF MS and Deeplex identifications were almost 100% concordant, with Deeplex providing subspecies-level detail for the *M. avium* complex and *M. abscessus*. Distinguishing *M. intracellulare* subsp. *chimaera* from *M. intracellulare* subsp. *intracellulare* and *M. abscessus* subsp. *abscessus* from *M. abscessus* subsp. *massiliense* has clinical value because expected drug responses and management can differ by subspecies (32, 33). This added resolution supports more targeted therapy and better infection-control decisions. However, subspecies-level assignments within these groups were based solely on Deeplex Myc-TB results and were not independently confirmed using orthogonal methods, which should be considered when interpreting these findings.

Three additional cultures showed discordant organism identification between Deeplex Myc-TB and either MALDI-TOF MS or conventional 16S sequencing on the initial testing run. Specifically, *M. heckeshornense* was misidentified by Deeplex Myc-TB as *M. abscessus* subsp. *abscessus* most likely due to potential carryover contamination from an adjacent sample containing that particular species. Failure to identify *M. heckeshornense* upon repeat testing despite adequate coverage depth may indicate that the current Deeplex library may not include sufficient entries to cover all geographical variations of this species. This may also be the case for the *M. porcinum* that was not identified by Deeplex Myc-TB but was confirmed by MALDI-TOF MS with a score of >2.0. These discrepant results highlight that such cases may require additional laboratory workup and are not fully captured by single-run comparisons. Finally, there was one instance where an isolate was identified by MALDI-TOF MS as *M. peregrinum* but not identified by Deeplex Myc-TB, which may underscore limitations in Deeplex species-level assignment for certain NTM taxa.

The present study has several limitations. It is a single-center snapshot from a tertiary referral institution. The patient population served by the Johns Hopkins Hospital and the complexity of submitted specimens may not capture the diversity of patients and laboratories nationwide; hence, the observed organism frequencies and resistance patterns may not generalize to other U.S. regions or healthcare settings. Additionally, demographic and clinical data for the contributing isolates were not collected, limiting assessment and preventing the correlation of genotypes with patient factors or clinical outcomes. Regarding mycobacterial identification at the subspecies level, no comparator assay was available to confirm subspecies identification within the MAC or for *M. abscessus*; thus, no conclusive statement may be made as a result of this study with respect to the accuracy of these particular identifications. Phenotypic interpretation was incomplete for some drugs because of panel design or the absence of CLSI breakpoints, which constrained categorical assignment. Although Deeplex Myc-TB detects many validated MTBC resistance markers, coverage for some newer agents, including non-*Rv0678* mutations for BDQ as well as pretomanid, remains incomplete, and predictive frameworks for NTM drug resistance continue to evolve. While Deeplex Myc-TB can identify the most common members of the MTBC (specifically *M. tuberculosis*, *M. bovis,* and *M. bovis* BCG) to the species level, species-level resolution may be reduced when sequencing depth is limited. This generally does not change empiric first-line therapy but may hinder recognition of species-specific features (e.g., intrinsically pyrazinamide-resistant *M. bovis*) and related epidemiologic investigations.

At the epidemiologic level, Deeplex Myc-TB can deepen understanding of regional mycobacterial diversity and resistance evolution by providing lineage, spoligotype, and heteroresistance data for MTBC. Documenting both known and emerging variants can strengthen genomic surveillance and support harmonization of molecular data across laboratories. Integration of the results into national and international mutation databases can improve early detection of drug resistance and inform public health responses to tuberculosis and NTM infections. However, as a targeted next-generation sequencing platform, Deeplex Myc-TB requires specialized infrastructure, technical and bioinformatics support, and sustained investment in reagents and quality management. These requirements may limit implementation in many low- and middle-income countries, where TB burden is highest, and laboratory resources are constrained. This highlights the need for context-appropriate deployment alongside simpler molecular and phenotypic tests.

Future work should focus on expanding resistance-marker coverage, especially for newer anti-TB agents, improving performance in low-bacillary specimens, and linking genotypes with clinical outcomes to calibrate predictive models. Harmonized workflows that integrate sequencing with targeted phenotypic testing will improve diagnostic reliability, shorten time to effective therapy, and enhance the utility of laboratory reports for clinicians. Implementation studies in high-burden, resource-limited settings will also be essential to define where tNGS adds the most value within tiered diagnostic networks.

In summary, Deeplex Myc-TB provides a robust, high-resolution platform for simultaneous detection of MTBC and NTM. Its ability to identify MTBC isolates, predict genotypic drug resistance, and accurately classify NTM species while also providing subspecies-level information underscores its value as a comprehensive diagnostic and surveillance tool in clinical microbiology. However, this platform, while it adds to our diagnostic catalog, by no means negates the requirement for pDST to continue as a comparator, especially in light of uncharacterized mutations for which few data are available. By combining high-resolution species identification with genotypic resistance calls that align with phenotypic results, Deeplex Myc-TB can shorten the time to effective therapy, strengthen molecular surveillance, and advance precision diagnosis and resistance monitoring for mycobacterial infections.

## SUPPLEMENTAL MATERIAL

Table S1 jcm.00082-26-s0001.docx MIC ranges for selected antitubercular agents tested by the Sensititre MYCOTB pDST panel and Deeplex Myc-TB.

ASM does not own the copyrights to Supplemental Material that may be linked to, or accessed through, an article. The authors have granted ASM a non-exclusive, world-wide license to publish the Supplemental Material files. Please contact the corresponding author directly for reuse.
