# Supplementary material for: Evaluation of the deeplex Myc-TB-targeted next-generation sequencing assay for mycobacterial identification and drug resistance detection
Source: J Clin Microbiol. 2026 May 29;64(7):e00082-26. doi: 10.1128/jcm.00082-26 (PMC13343837; doi:10.1128/jcm.00082-26)
Supplement: Table S1 — MIC ranges for selected antitubercular agents tested by the Sensititre MYCOTB pDST panel and Deeplex Myc-TB. [file jcm.00082-26-s0001.docx]

Supplementary Table 1. MIC ranges for selected antitubercular agents tested by the Sensititre MYCOTB pDST panel and Deeplex® Myc-TB

| **Drug** | **Observed Sensititre**  **MYCOTB pDST (MIC)** | **WHO MGIT critical concentration^1,2^** | **Deeplex® Myc-TB**  **variant detection** |
| --- | --- | --- | --- |
| BDQ | Not tested | 1 µg/mL | No variant detected |
| CFZ | Not tested | 1 µg/mL | No variant detected |
| LZD | Not tested | 1 µg/mL | Uncharacterized variant  detected: *rrl* (G2399A) |
| MXF | 0.12 µg/mL – 1.0 µg/mL | 0.25 µg/mL | Uncharacterized variant  detected for FQ: *gyrA* (V55M) |
| LFX | Not tested | 1 µg/mL |  |
| AMK | 0.12 µg/mL – 0.5 µg/mL | 2 µg/mL | No variant detected |
| KAN | 0.6 µg/mL – 2.5 µg/mL | 2.5 µg/mL | No variant detected |
| STM | 0.25 µg/mL – 4.0 µg/mL | 1 µg/mL | Characterized variants detected: *rpsL* (K43R), *gidB* (delG), *gidB* (del) |

BDQ – Bedaquiline; CFZ – Clofazimine; LZD – Linezolid; MXF – Moxifloxacin; LFX – Levofloxacin; AMK – Amikacin; KAN – Kanamycin;

STM – Streptomycin; FQ – Fluoroquinolone; pDST – Phenotypic drug susceptibility testing; MIC – Minimum inhibitory concentration

**^1^World Health Organization.** 2018. Technical report on critical concentrations for drug susceptibility testing of medicines used in the treatment of drug-resistant tuberculosis. World Health Organization, Geneva, Switzerland.

**^2^World Health Organization.** 2024. WHO consolidated guidelines on tuberculosis: Module 3—Diagnosis: Rapid diagnostics for tuberculosis detection. World Health Organization, Geneva, Switzerland.
